# Roles of *HOTAIR* Long Non-coding RNA in Gliomas and Other CNS Disorders

**DOI:** 10.1007/s10571-024-01455-8

**Published:** 2024-02-16

**Authors:** Faraz Ahmad, Ravi Sudesh, A. Toufeeq Ahmed, Shafiul Haque

**Affiliations:** 1 Department of Biotechnology, School of Bio Sciences (SBST), Vellore Institute of Technology (VIT), Vellore, 632014 India; 2 Department of Biomedical Sciences, School of Bio Sciences (SBST), Vellore Institute of Technology (VIT), Vellore, 632014 India; 3 Research and Scientific Studies Unit, College of Nursing and Health Sciences, Jazan University, Jazan, 45142 Saudi Arabia; 4 Gilbert and Rose-Marie Chagoury School of Medicine, Lebanese American University, Beirut, 1102 2801 Lebanon; 5 Centre of Medical and Bio-Allied Health Sciences Research, Ajman University, Ajman, 13306 United Arab Emirates

**Keywords:** Brain cancer, Alzheimer’s disease, Parkinson’s disease, Sponging, Neuropsychiatric diseases, Traumatic brain injury

## Abstract

HOX transcript antisense intergenic RNA (*HOTAIR*) is a long non-coding RNA (lncRNA) which is increasingly being perceived as a tremendous molecular mediator of brain pathophysiology at multiple levels. Epigenetic regulation of target gene expression carried out by *HOTAIR* is thorough modulation of chromatin modifiers; histone methyltransferase polycomb repressive complex 2 (PRC2) and histone demethylase lysine-specific demethylase 1 (LSD1). Incidentally, *HOTAIR* was the first lncRNA shown to elicit sponging of specific microRNA (miRNA or miR) species in a trans-acting manner. It has been extensively studied in various cancers, including gliomas and is regarded as a prominent pro-tumorigenic and pro-oncogenic lncRNA. Indeed, the expression of *HOTAIR* may serve as glioma grade predictor and prognostic biomarker. The objective of this timely review is not only to outline the multifaceted pathogenic roles of *HOTAIR* in the development and pathophysiology of gliomas and brain cancers, but also to delineate the research findings implicating it as a critical regulator of overall brain pathophysiology. While the major focus is on neuro-oncology, wherein *HOTAIR* represents a particularly potent underlying pathogenic player and a suitable therapeutic target, mechanisms underlying the regulatory actions of *HOTAIR* in neurodegeneration, traumatic, hypoxic and ischemic brain injuries, and neuropsychiatric disorders are also presented.

**Graphical Abstract:**

HOTAIR-mediated epigenetic DNA regulation and molecular sponging of target miRNAs. While the 5′ end of HOTAIR regulates the H3K27 trimethylation activity of the catalytic subunit enhancer of Zeste homolog 2 (EZH2) of the polycomb repressive complex 2 (PRC2), its 3′ end modulates the H3K4 demethylation activity of lysine-specific demethylase 1 (LSD1). HOTAIR also binds to and competitively inhibits the functions of target miRNAs, altering the expression of downstream genes. 
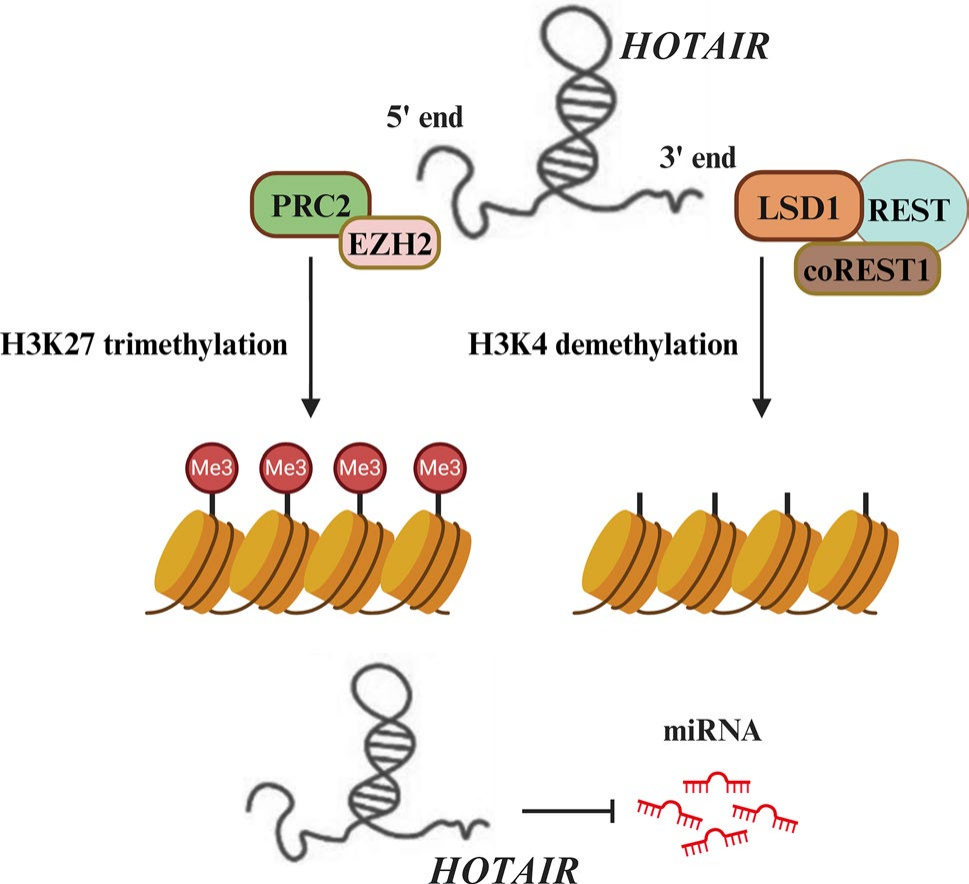

## Introduction

Long non-coding RNAs (lncRNAs) are RNA species with sizes greater than 200 nucleotides that are not translated into polypeptides. LncRNAs are widely localized at multiple genomic regions; however, they elicit considerable versatility in their biogenesis, cell- and tissue-specific expression patterns, and pathophysiological functions (Bridges et al. 2021). The latest version of Gencode 43 (2022) catalog reports 19,928 lncRNA genes which produce a whopping cumulative total of 58,023 transcripts. The multifaceted cellular functions of lncRNAs are due to their ability to interact with diverse categories of biomolecules, including DNA, mRNA, proteins, and even other non-coding RNAs (e.g., microRNAs or miRNAs) (Herman et al. 2022). One of the prominent mechanisms by which lncRNAs influence cellular functions is by affecting chromatin organization and remodeling, transcription mechanisms, and splicing and export of mRNA species. Interestingly, lncRNAs also regulate the cytoplasmic stability, localization, miRNA-targeting and translation of other RNAs by acting as “molecular sponges” (Borkiewicz et al. 2021). Further, lncRNA species have been reported to induce posttranslational modifications of proteinaceous components (Srinivas et al. 2023). Cells of the central nervous system (CNS) with all their morpho-functional complexities are tremendous targets of lncRNA-mediated alterations in pathophysiological processes (Cuevas-Diaz Duran et al. 2019). Both neurons and glia show abundant expression of various lncRNAs. Indeed, several recent studies have identified prominent roles of different lncRNA species in brain function modulation. LncRNAs have been implicated as intricate and dynamic regulators of multiple aspects of CNS pathophysiology (Cuevas-Diaz Duran et al. 2019), including tumorigenesis (DeSouza et al. 2021; Kim et al. 2021). Because of their multifaceted roles in altering neuronal pathophysiological trajectories, lncRNAs have been envisioned as promising biotargets for diagnosis, prognosis and treatment of multiple neurological disorders (Yang et al. 2021).

**Fig. 1:**
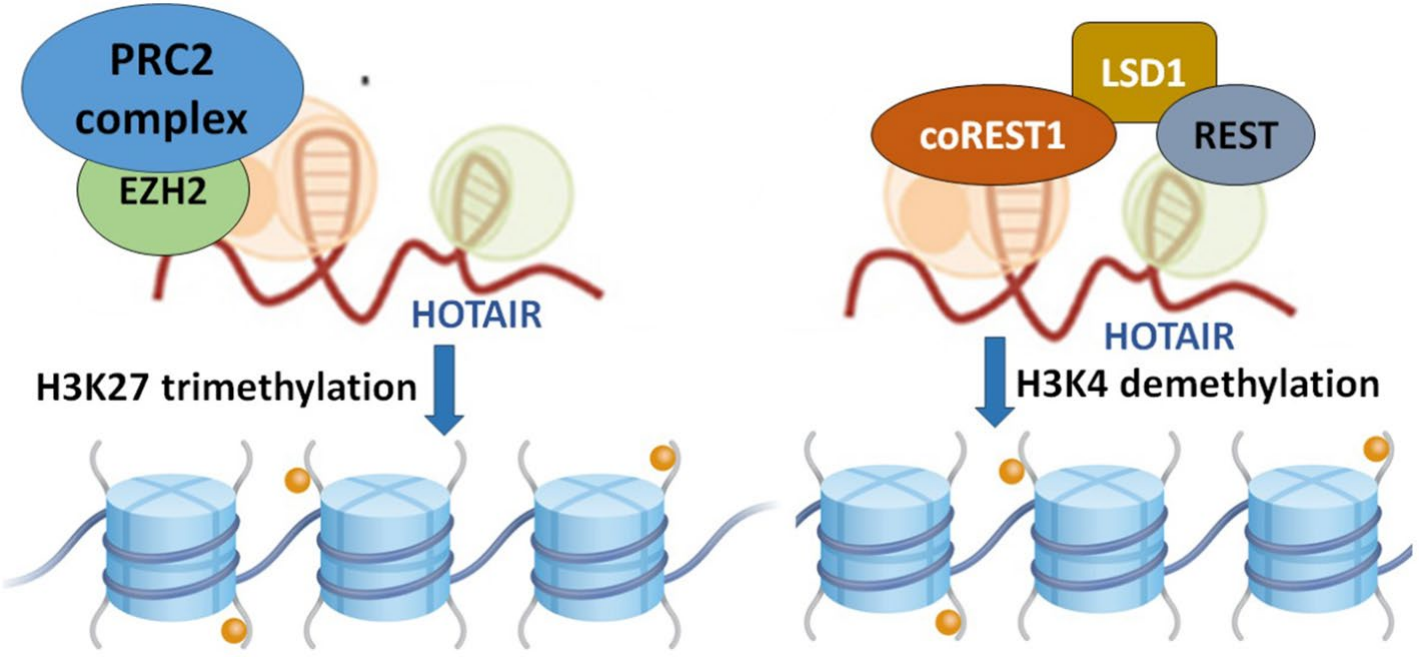
The mechanism of epigenetic regulation mediated by HOTAIR lncRNA. 5′ end of HOTAIR is involved in the binding and regulation of H3K27 trimethylation activity of the polycomb repressive complex 2 (PRC2) via the catalytic subunit EZH2. On the other hand, 3′ end of HOTAIR modulates the H3K4 demethylation activity of lysine-specific demethylase 1 (LSD1)—RE1 silencing transcription factor (REST)—REST corepressor 1 (coREST1)

*HOTAIR* (aka HOX transcript antisense intergenic RNA) is generated from the antisense strand of the *HOXC* gene, which is localized on chromosome 12q13.13 (Price et al. 2021). *HOTAIR* transcript consists of 6 exons and 2158 nucleotides, and is spliced and polyadenylated (Xin et al. 2021). It was one of the first lncRNAs reported to elicit trans-silencing of genes. Mechanistically, *HOTAIR* regulates the functions of histone methyltransferase, polycomb repressive complex 2 (PRC2) via its interaction with enhancer of Zeste homolog 2 (EZH2), PRC2’s catalytic subunit (Fig. 1). Specifically, *HOTAIR* modulates trimethylation activity (at lysine-27 of histone H3; H3K27me3) of PRC2/EZH2, resulting in epigenetic transcriptional repression of the target genes (Rinn et al. 2007). Further, *HOTAIR* also forms a physical scaffold and aids complex formation of lysine-specific demethylase 1 (LSD1), repressor element 1 silencing transcription factor (REST), and REST corepressor 1 (CoREST1). This results in modulation of the latter’s ability to demethylate lysine-4 of histone H3; H3K4me2 (Tsai et al. 2010). Studies have indicated that while the 5′ domain of *HOTAIR* binds to and regulates EZH2/PRC2, its 3′ domain is implicated in the regulation of LSD1-REST-CoREST functions. Together, these mechanisms robustly contribute to *HOTAIR*’s ability to influence epigenetic statuses of numerous target genes (Cai et al. 2014).

*HOTAIR* has been proposed to influence diverse pathways in the pathogeneses of multiple types of inflammatory dysfunctions and malignancies [reviewed in Ghafouri-Fard et al. 2021; Price et al. 2021)]. Elevations in the expression and hyperactivation of *HOTAIR*, and the consequent retargeting of PRC2 and LSD1-REST-coREST1 are thought to be associated with dysregulated epigenetic statuses of underlying genes and aggravation of tumor cell invasiveness and metastases. Conversely, measures directed at repression of *HOTAIR* expression and activity are related to attenuation of tumorigenesis and cancer progression (Xin et al. 2021). Hence, *HOTAIR* may be envisioned as a relevant biomarker and therapeutic target in oncological pathogeneses (Lu et al. 2017). Not surprisingly, elevations in *HOTAIR* levels and the associated detrimental effects on chromatin modification, gene regulation, transcription, RNA processing, and post-transcriptional regulation of downstream genes have been reported to be associated with high tumor grades and severities, as well as poor survival in several cancer types (Hajjari and Salavaty 2015). At the molecular and cellular levels, *HOTAIR* has been shown to promote tumorigenic pathways via alteration of a variety of signaling cascades (Chen et al. 2021). In fact, *HOTAIR* represents one of the most prominent and extensively studied lncRNAs which are often found to be hyperactivated in multiple human tumor types, including breast, lung, gastric, colorectal, cervical, and brain cancers.

The objective of the current review is to comprehensively compile the research data implicating *HOTAIR* lncRNA in a plethora of pathophysiological aspects of brain cancers, particularly gliomas. Relevances of *HOTAIR* as a prognostic marker and therapeutic target in gliomas and other brain cancers are discussed in detail. Further, the authors summarize the roles of *HOTAIR* in the pathogeneses of other neuronal disorders, including traumatic brain injury (TBI), psychiatric conditions, and neurodegenerative diseases.

## Overview of Tumorigenic Functions of *HOTAIR*

### MiRNA Cross Talk

**Fig. 2:**
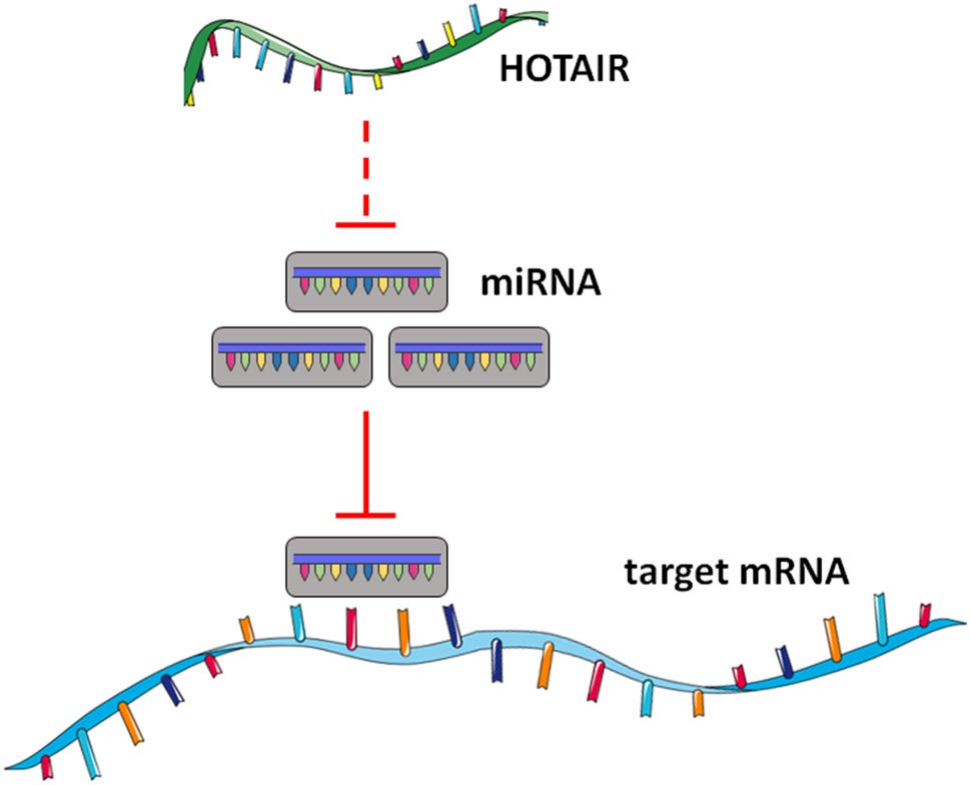
HOTAIR as a molecular sponge for miRNAs. HOTAIR acts as a competing endogenous (ceRNA) by binding and inhibiting specific miRNAs, thereby removing the expressional repression of downstream target mRNA transcripts

A wide variety of lncRNA species and their competing endogenous RNA (ceRNA) actions against miRNAs are implicated in the pathophysiology of multiple CNS disorders (Moreno-García et al. 2020; Luo et al. 2021). Incidentally, *HOTAIR* was one of the first lncRNAs discovered to serve as a “molecular sponge” for inhibiting the activity of multiple miRNA species (Fig. 2). This ability of *HOTAIR* to act as a ceRNA allows it to abolish availability of target miRNA species for regulating the translation of downstream mRNAs (also see “*HOTAIR*-Mediated Trans-Silencing of miRNAs in Glioma Pathogeneses” section). For example, HOTAIR competitively binds to miR-20a-5p to block the latter’s interaction with high mobility group A2 (HMGA2) mRNA, thereby increasing the protein levels of HMGA2, and resulting in aggravation of tumorigenicity of breast cancer cells (Zhao et al. 2018). Similarly, HOTAIR has been reported to sequester miR-129-5p, causing pathogenic increases in the levels of Frizzled 7 (FZD7) (Wu et al. 2021).

### Epithelial-Mesenchymal Transition (EMT)

EMT is a multistep process associated with forfeit of epithelial characteristics of cells, and acquirement of mesenchymal attributes, such as cellular motility, migration, and invasiveness. The transdifferentiation pathway of EMT is particularly crucial for tumorigenesis, and tumor cell metastasis and migration. Studies indicate that *HOTAIR* critically influences EMT at multiple levels. Correspondingly, *HOTAIR*-mediated induction of EMT has been proposed in the pathogeneses of multiple cancers (Amicone et al. 2023). For instance, transforming growth factor beta 1 (TGF-β1)-induced pathogenic overactivation of *HOTAIR* robustly triggers EMT in colon cancer cells, and repression of *HOTAIR* correspondingly results in diminution of EMT and metastatic abilities of these cells (Pádua Alves et al. 2013). Similarly, overexpression of *HOTAIR* in oral squamous cell carcinomas is implicated in increased EMT and invasiveness of tumor cells, while its ablation rescues cellular stemness and tumorigenicity of oral carcinoma cells (Lu et al. 2017). Studies with a dominant negative mutant of *HOTAIR* in TGF-β-induced EMT have confirmed its ability to bind to and regulate the biological functions of Snail protein (a master regulator of EMT pathway) in the repression of target epithelial genes (Battistelli et al. 2021).

## *HOTAIR* and Gliomas and Other Brain Cancers

Among the primary brain cancers, gliomas represent the most prevalent and heterogeneous cancer types, and are extremely difficult to diagnose, classify, and treat. In recent years, there has been a tremendous surge in the number of studies focused at understanding the pathogenic and protective actions of different lncRNA species (including *HOTAIR*) in the development of gliomas (Angelopoulou et al. 2020; Cantile et al. 2021).

### HOTAIR as a Predictor of Glioma Grade and as a Prognostic Marker

Zhang et al. (2013) were among the first researchers to propose the involvement of *HOTAIR* in the pathogenesis of gliomas. Further, based upon their observations of its preferential expression in classical and mesenchymal gliomas, rather than the neural and proneural subtypes, they advocated that *HOTAIR* could serve as a relevant biomarker for differentiating gliomas on the bases of the different gene expression-based molecular subtypes. Moreover, they proposed *HOTAIR* as a potential marker for tumor grade as its expression was found to be strongly and positively correlated with the tumor grade and severity. Statistical analyses based on correlational Kaplan–Meier survival curve between its expression and survival (hazard ratio; HR 2.933) indicated that *HOTAIR* lncRNA is a potential prognostic biomarker for predicting survival rates in glioma clinical cases. Mechanistically, employing siRNA-mediated repression of *HOTAIR* in glioblastoma cell lines (LN229 and U87), the authors found that it regulates cell cycle progression via its inhibitory actions on signaling through transcription factor E2F1, cyclin D1 and E, and cyclin-dependent kinases CDK2 and 4; and via its stimulatory actions on cell cycle proteins, p16 and p21. Lastly, knockdown of *HOTAIR* under in vivo settings in glioma harboring mice was shown to robustly reduce tumor burden (Zhang et al. 2013). In their follow-up study, the research group identified EZH2/PRC2 as the major molecular target of *HOTAIR*-mediated pro-oncogenic effects (Zhang et al. 2015). Further, gene ontology (GO) analyses of known targets of *HOTAIR* confirmed the involvement of physiological cascades of cell proliferation, organization and biogenesis, DNA and RNA metabolism, and cell cycle progression (Zhang et al. 2015). Small nucleolar RNA, C/D box small nucleolar RNA 76 (SNORD76) was identified as another molecular target of *HOTAIR* signaling. Thus, *HOTAIR* robustly reduced the expression of SNORD76, and involvement of HOTAIR/SNORD76 signaling in glioblastoma pathogeneses was confirmed both in nude mice with ectopically transplanted U87 cells, and in brain tumor specimens from human subjects (Chen et al. 2015). Further studies indicated that the tumorigenic actions of *HOTAIR* in gliomas additionally rely on its ability to suppress programmed cell death protein 4 (PDCD4) in a PRC2-dependent manner (Chen et al. 2016). More recently, it was reported that the pro-oncological mediations of *HOTAIR* likely depend on hyperactivation of β-catenin cascade, possibly in a Nemo-like kinase (NLK)-dependent manner (Zhu et al. 2021).

Analyses of *HOTAIR* gene expression, methylation status, and copy number in human glioma cases, such as isocitrate dehydrogenase (IDH)-wild type glioblastoma, support the hypothesis of the former’s positive association with glioma grades. Importantly, *HOTAIR* was confirmed as a prognostic marker for gliomas since patients eliciting its high levels were observed to have significantly decreased survival (Xavier-Magalhães et al. 2018). Further, robust association of the expression of *HOTAIR* with the levels of homeobox protein HOXA9 was observed in glioma tissues, particularly from clinical subjects of higher-grade gliomas. Subsequent analyses using chromatin immunoprecipitation (ChIP)-qPCR methods indicated direct binding of HOXA9 to the promoter of the *HOTAIR* gene (Xavier-Magalhães et al. 2018). In concurrence with these results, upon an Arraystar Human lncRNA Microarray-based investigation of the differential expressions of over 33,000 lncRNAs in glioblastoma cases compared to controls, *HOTAIR* was recognized as a primary lncRNA species with significantly increased expression in glioblastoma (Yan et al. 2015). In consistence, a strong positive association of *HOTAIR* expression with glioma grades was observed in Chinese clinical cases after adjusting for gender and age (Zhao et al. 2019). Similarly, RNA-seq data from Chinese clinical cases of different grades of gliomas indicated *HOTAIR* as the top upregulated lncRNA, possibly as a consequence of alterations in its epigenetic DNA methylation pattern (Li et al. 2019b). In contrast, a study reported diminished expression of *HOTAIR* lncRNA in U118, MG-U87, and MG-LN18 glioblastoma cells; however, it used human parental brain cancer stem cell line as controls (Balci et al. 2016).

An interesting study by Lei et al. (2018) used total RNA sequencing data from 152 glioblastoma subjects from The Cancer Genome Atlas (TCGA) and identified *HOTAIR* as one of the prominent members in a group of 9 lncRNAs that could potentially be used as a prognostic biomarker set for glioblastoma as well as for predicting patient survival. Further, bioinformatic analyses indicated the possible involvement of several downstream target genes central to a plethora of signaling pathways, such as cell–cell interaction, neurotransmitter physiology, and calcium signaling (Lei et al. 2018). While actual experimental data are needed to confirm these findings and to identify the exact molecular players and pathways linking *HOTAIR* and glioma development, other in silico studies have also been conducted to propose the multimodal tumor-promoting activities of *HOTAIR* in gliomas. Using the Chinese Glioma Genome Atlas, Huang et al. (2017) identified *HOTAIR* as a master regulator of an mRNA network of 18 genes controlling cell cycle progression in the promotion of glioma cell proliferation. These predictions were confirmed experimentally using an in vivo rodent model with glioblastoma xenograft, wherein the authors also observed robust anti-glioma effects of strategies to knockdown *HOTAIR* expression (Huang et al. 2017).

Computational analyses of lncRNAs as regulators of transcription factor-gene target relationships have resulted in the identification of several lncRNA-transcription factor-gene target axes with a close association with glioblastoma progression, including *HOTAIR*-MAX interacting protein 1CD 58-protein kinase C epsilon (HOTAIR-MXI 1-CD 58-PRKCE) and *HOTAIR*-activating transcription factor 5-neural cell adhesion molecule 1 (HOTAIR-ATF 5-NCAM 1) triplets (Li et al. 2016b). These results are in concurrence with a study which used data from TCGA database, and proposed *HOTAIR*, *DLEU1,* and *LOC00132111* as the lncRNAs with the most significant associations with overall survival of glioma subjects (Lv et al. 2020). Another study based upon data from TCGA database proposed *HOTAIR* as a prominent lncRNA with robust elevations in the expression levels in glioblastoma subjects (Li et al. 2016a). Interestingly, data extracted from TCGA and Gene Expression Omnibus databases for clinical cases of glioblastomas and low-grade gliomas (LGGs) identified *HOTAIR* as among the 13 lncRNAs that could potentially distinguish glioblastomas from LGGs (Li et al. 2021).

In conclusion, these data suggest that *HOTAIR* expression may be a relevant and potent prognosticator of glioma progression, severity, and grade type, either alone or in combination with other biomarkers. Indeed, meta-analysis of systematically reviewed primary data supports this hypothesis (Zhou et al. 2018). In addition, *HOTAIR* expression as a negative prognostic predictor of poor outcomes in glioma patients raises the possibility of its potential utility as a biotarget for oncological therapies, particularly in gliomas (“*HOTAIR*-Based Diagnostic and Prognostic Biomarkers from Peripheral Samples” section).

### *HOTAIR*-Mediated Trans-Silencing of miRNAs in Glioma Pathogeneses

As discussed, in addition to the epigenetic mediations, *HOTAIR* is a prominent component of the ceRNA networks because of its trans-silencing (molecular sponging) ability against multiple miRNAs. This contributes as another interesting facet of *HOTAIR*-dependent pro-tumorigenic gene expressional effects in gliomas. Below, we delineate, in detail the results from the studies which have focused on molecular sponging-reliant effects of *HOTAIR* against multiple target miRNA species in glioma pathophysiology (Table 1).

**Table 1:** Sponging targets of *HOTAIR* in glioma pathogenesis

miRNA target	Signaling axis	Pathophysiological effect	References
miR-141	*HOTAIR*/miR-141/SKA2	*HOTAIR* expression was negatively linked with miR-141 levels in glioma tissues, resulting in aggravated glioma cell proliferation and metastasis; knockdown of *HOTAIR* culminated in reduced tumor load in in vivo xenograft tumor model in mice	Bian et al. (2016)
miR-326	*HOTAIR*/miR-326/FGF-1	Elevated *HOTAIR* levels in glioma tissues and cell lines correlated with attenuated FGF-1 levels and induction of oncogenic pathways via PI3K/Akt and MEK 1/2 pathways; *HOTAIR* knockdown resulted in increased survival of mice challenged with tumors	Ke et al. (2015)
miR-148b-3p	*HOTAIR*/miR-148b-3p	Increased *HOTAIR* expression in glioma tissues and cell lines; silencing of HOTAIR caused reduced survival, growth and proliferation	Wang et al. (2016)
	*HOTAIR*/miR-148b/3p-USF-1	Elevated levels of *HOTAIR* in glioma microvascular endothelial cells resulting in dysregulation of blood-tumor barrier (BTB); *HOTAIR* knockdown increased BTB permeability in complementation with repression of tight junction-associated proteins	Sa et al. (2017)
miR-301a-3p	*HOTAIR*/miR-301-3p/FOSL 1	TRMP-7-mediated hyperactivation of *HOTAIR* in glioma cells was related to heighted cellular proliferation, invasion, and metastasis in a FOSL 1-dependent manner	Guo et al. (2022)
miR-126-5p	*HOTAIR*/miR-126-5p/GLS	Significantly upregulated levels of *HOTAIR* expression in high-grade glioma tissues, compared to low-grade ones; *HOTAIR* hyperactivation resulted in deficits in glutamine metabolism, aberrant angiogenesis, and aggravated chemoresistance	Liu et al. (2018a)
miR-15b	*HOTAIR*/miR-15b	*HOTAIR* upregulation in glioma cells was related to altered miR-15b-p53 signaling, resulting in enhanced survival, proliferation, and invasiveness of the cancerous cells	Sun et al. (2018)
miR-218	*HOTAIR*/miR-218/PDE7A	Increased cytoplasmic *HOTAIR* expression in glioma tissues and cell lines was linked with increased survival, growth, proliferation, and malignancy; conversely its repression induced cellular death and repress tumorigenicity	Wei et al. (2020)
miR-219	*HOTAIR*/miR-219	siRNA-induced repression of *HOTAIR* in glioblastoma U87 cells resulted in inhibition of cellular proliferation and induction of apoptosis	Li and Guan (2020)

Data from both human glioma tissues and U251 and U87 cells indicate that *HOTAIR* is responsible for trans-silencing of miR-141. Interestingly, miR-141 may directly bind to and repress the expression of *HOTAIR*. Such negative regulation of *HOTAIR* functions mediated by miR-141, possibly dependent on signaling through the spindle and kinetochore-associated protein 2 (SKA2), has been proposed as the underlying cause of its tumor suppression activities. Indeed, alterations in the *HOTAIR*-miR-141-SKA2 signaling axis by either knockdown of *HOTAIR* lncRNA or overexpression of miR-141 results in inhibition of growth of ectopic tumor xenografts in mice (Bian et al. 2016). Another molecular sponge of *HOTAIR* implicated in glioma pathogenesis is miR-326 (Ke et al. 2015). Thus, increases in the levels of *HOTAIR* in glioma tissues and in vitro tumor cells have been found to be associated with reduced expression of miR-326. Mechanistically, *HOTAIR*-mediated sponging of miR-326 promotes tumorigenesis by hyperactivating fibroblast growth factor 1 (FGF-1) signaling, resulting in aberrant stimulation of downstream phosphoinositide 3-kinase (PI3K)-Akt and mitogen-activated protein kinases MAPK1/2 cell signaling pathways. Further, inhibition of *HOTAIR* and the consequent removal of miR-326 silencing were found to protect nude mice challenged with orthotopic tumor transplantation (Ke et al. 2015). Experimental data from A172 glioma cells identified miR-148b-3p as yet another direct mediator of *HOTAIR* signaling in gliomas. *HOTAIR* appeared to cause sponging of miR-148b-3p, and silencing the former resulted in diminution of cell survival, proliferative, and metastasis of tumor cells in a miR-148b-3p-dependent manner (Wang et al. 2016). Interestingly, *HOTAIR*-miR-148b-3p signaling has also been implicated in the dysregulation of blood-tumor barrier in gliomas via the mediation of the upstream stimulatory factor 1 (USF 1) pathway (Sa et al. 2017). Hence, *HOTAIR* levels were observed to be significantly increased in a glioma model of blood-tumor barrier constructed by co-culture of human cerebral microvascular endothelial hCMEC/D3 cells and U87 cells, in complementation with reduced expression of miR-148b-3p and pathogenic increments in USF 1 function. Proteinaceous components of the tight junctions of microvascular endothelial cells, zonula occludens 1 (ZO 1), occludin, and claudin 5 were also found to be altered. Further, *HOTAIR* knockdown resulted in normalized miR-148b-3p levels, which in turn promoted rescue of the alterations in USF-1 and tight junction proteins (Sa et al. 2017).

MiR-301a-3p and its downstream target, pro-tumorigenic transcription factor, Fos like 1 (FOSL 1) represent another set of mediators of *HOTAIR*’s actions in glioma pathogenesis. Thus, transient receptor potential melastatin 7 (TRPM7) channels-induced and *HOTAIR* upregulation-mediated silencing of miR-301a-3p was found to culminate into significant elevations in the levels of FOSL 1 in glioma tissues (Guo et al. 2022). Similarly, miR-126-5p-glutaminase (GLS) axis has been proposed as a prominent mediator of the pro-oncological actions of *HOTAIR* (Liu et al. 2018a). *HOTAIR* as a ceRNA against miR-126-5p promotes GLS expression, as suggested by clinical data from human subjects of astrocytomas, oligodendrogliomas, and glioblastomas; as well as from exploratory studies in glioma cell lines, U87 and U251. Thus, *HOTAIR*-miR-126-5p-GLS signaling may have significant effects on multiple aspects of glutamine metabolism, aberrant angiogenesis, cellular invasiveness, and temozolomide resistance (see also “HOTAIR as a Mediator of Chemoresistance in Gliomas” section) in glioma pathophysiology (Liu et al. 2018a).

MiR-15b-p53 regulatory loop which is implicated in regulating growth, proliferation, and invasiveness of glioma cells has also been reported to be under the modulatory control of *HOTAIR*-mediated trans-silencing. It has been observed that induction of miR-15b and p53 promotes apoptosis and inhibits proliferation of U87 cells, while *HOTAIR* hyperactivity conversely results in increased proliferative and invasive properties of these cells (Sun et al. 2018). MiR-218-phosphodiesterase 7A (PDE7A) signaling axis is another mediator linking hyperactivation of *HOTAIR* and glioma pathogeneses. Hence, glioma tissue samples and cell lines elicit aberrantly elevated levels of *HOTAIR* in complementation with significantly repressed levels of miR-218. Direct proof of the involvement of the *HOTAIR*-miR-218-PDE7A loop in gliomas has been confirmed by both sh-RNA-mediated silencing of *HOTAIR* and overexpression of miR-218 (Wei et al. 2020). Another recently discovered sponging target of *HOTAIR* with relevance for gliomas is miR-219, as confirmed by siRNA-mediated silencing of *HOTAIR* in U87 cells (Li and Guan 2020).

### HOTAIR as a Mediator of Chemoresistance in Gliomas

In addition to the pro-tumorigenic effects, several recent studies indicate that *HOTAIR* signaling plays prominent roles in conferring chemoresistance to tumor cells (Singh et al. 2020). Pharmacological agents have been shown to be rendered severely ineffective because of the hyperactivation of HOTAIR signaling in gliomas. For instance, Yuan et al. (2020) showed that *HOTAIR*-miR-519a-3p/ribonucleoside diphosphate reductase subunit M1 (RRM1) signaling has been implicated in conferring resistance to human glioblastoma cells, A172 and LN229 against temozolomide. *HOTAIR* expression was found to be significantly increased in temozolomide resistant glioblastoma cells, and its silencing culminated into regularized vulnerability of the cell and in vivo xenograft tumors to temozolomide. Correspondingly, induction of temozolomide resistance was found to be accorded via *HOTAIR*-containing exosomes obtained from the temozolomide resistant glioblastoma cells. Further, based upon data from clinical cases of glioblastoma with and without temozolomide resistance, the authors advocated the employment of serum exosomal *HOTAIR* expression as a diagnostic biomarker for temozolomide sensitivity of glioblastomas (Yuan et al. 2020). This hypothesis seems plausible since extracellular vesicles carrying lncRNAs, among other payload biomolecules, are agents for altering the pathogenic cascades of gliomas and other cancers at multiple levels. Indeed, exposure of serum-derived extracellular vesicles from glioblastoma subjects containing high abundances of *HOTAIR* was found to robustly facilitate glioma cell proliferation, invasiveness, and temozolomide resistance both in vitro and in vivo (Wang et al. 2022a). Such extracellular vesicular *HOTAIR*-conferred temozolomide resistance may plausibly be due to attenuation of miR-526b-3p-mediated inhibition of regulator of programmed cell death, EVA 1.

Resistance of gliomas to temozolomide may manifest due to alterations in the *HOTAIR*-miR125 signaling axis, via hyperactivation of hexokinase 2 (HK2)-mediated glycolytic pathway in tumor cells (Zhang et al. 2020a). Thus, downregulation of *HOTAIR* was reported to normalization of HK-2 mRNA and protein expressions, and increased temozolomide susceptibility in vitro, in U87 cells, as well as in vivo, in immune-compromised with mice subcutaneous glioma cell xenograft. Lastly, regulatory elements of *HOTAIR* gene were observed to determine temozolomide resistance in U251 glioma cells, possibly via expressional modulation of distant genes, calcium binding, and coiled-coil domain 1 (*CALCOCO1*) and zinc finger CCCH-type containing 10 (*ZC3H10*) (Zhang et al. 2020c).

### *HOTAIR* in Other Brain Cancers

*HOTAIR* as a pathogenic mediator in pediatric brain tumors has been proposed by Chakravadhanula et al. (2014). They used nanoString platform for transcriptomic analysis in various pediatric brain tumors, and observed significant elevations in *HOTAIR* levels in atypical teratoid rhabdoid tumors (ATRTs), juvenile pilocytic astrocytomas (JPAs), and medulloblastomas. Ependymomas, on the other hand, were associated with markedly reduced *HOTAIR* expression (Chakravadhanula et al. 2014). Mechanistic studies in medulloblastoma cells, Daoy and D341 as well as medulloblastoma xenograft mice models to discern the exact molecular mechanisms/players behind the pro-tumorigenic actions of *HOTAIR* revealed possible involvement of the miR-483-3p-CDK4 axis (Zhao et al. 2020b). Other players implicated in the tumorigenic actions of *HOTAIR* in medulloblastoma pathogenesis include miR-1 and miR-206, both of which are its sponging targets, and have the capacity to modulate expression of zinc finger transcriptional repressor, Yin Yang 1 (YY1) (Zhang et al. 2020b). Indeed, elevated levels of *HOTAIR* and YY1 have been observed in tissues from clinical cases of human medulloblastoma, and in Daoy, D283 Med and D341 cell lines. Correspondingly, repression of *HOTAIR* was observed to alter miR-1/miR-206-YY1 signaling in favor of repression of medulloblastoma cell proliferation, migration and epithelial to mesenchymal transition (EMT), and induction of apoptosis (Zhang et al. 2020b). Lastly, RNA in situ hybridization (RNA-ISH) analyses in ependymoma tissues unveiled significant upregulation of the expression levels of *HOTAIR* lncRNA; particularly in spinal myxopapillary ependymoma (SMPE), when compared to non-ependymoma tumors of the spinal cord (Zheng et al. 2020). Further studies however are warrantied to establish *HOTAIR* as a specific diagnostic marker for spinal MPE.

### Genetic Variations of *HOTAIR* and Brain Cancers

*HOTAIR* SNPs have been proposed to be associated with the development of multiple pathological states, including neuropsychiatric conditions (“Neuropsychiatric Disorders” section) and multiple sclerosis (“Multiple Sclerosis (MS)” section), and various cancer types. One of the pioneering studies focused at evaluation of linkages between *HOTAIR* polymorphisms and glioma pathogenesis was performed by Xavier-Magalhães et al. (2017). Using Portuguese clinical cases of multiple forms of glioma and controls, they found significant associations of rs920778 CT and rs12826786 CT genotypes with the survival of subjects diagnosed with grade III anaplastic oligodendroglioma. However, no statistically significant differences were observed for distribution of these SNPs per se between the glioma cases and controls. Based upon these findings, the authors propounded the potential utilities of these SNPs as prognostic biomarkers for anaplastic oligodendroglioma (Xavier-Magalhães et al. 2017). Needless to say, future studies are warrantied to evaluate and ascertain the associations of other SNPs of *HOTAIR* in the development and prognosis of the different types of brain cancers and gliomas.

### *HOTAIR*-Based Diagnostic and Prognostic Biomarkers from Peripheral Samples

As already discussed, elevated expression of *HOTAIR* is a potent indicator of high grade/severity, poor prognosis, and reduced survival in gliomas. Interestingly, Ren et al. have proposed the use of radiolabeled antisense oligonucleotide probes against *HOTAIR* in liposomal encapsulations for in vivo real-time imaging of *HOTAIR*-positive gliomas (Ren et al. 2022).

Liquid biopsy-based peripheral biomarkers represent a minimally invasive and effective source of prognostic and diagnostic information in oncology (Shah et al. 2022), particularly for gliomas and other CNS tumors. Indeed, lncRNAs from peripheral circulations represent a relevant category of prognostic platforms for gliomas (Li et al. 2016a). For instance, assessment of the serum levels of known pro-tumorigenic lncRNAs (including *HOTAIR*) of 106 clinical cases diagnosed with primary glioblastoma was undertaken by Shen et al. (2018). Regarding *HOTAIR*, the authors found a strong positive association with recurrence and progression of metastases (adjusted hazard ratio of 1.82), and increased likelihood of mortality (adjusted hazard ratio of 2.04). Further, serum levels of *GAS5* lncRNA were found to be linked with diminished tumor recurrence and progression, indicating the possible utilities of circulating *HOTAIR* and *GAS5* in serving as potential reciprocal predictors of disease prognosis and survival in glioblastomas (Shen et al. 2018). Serum-derived exosomes as peripheral prognostic and diagnostic platforms for gliomas, specifically for the evaluation of *HOTAIR* expression have also been proposed in recent studies (Malissovas et al. 2019). Thus, Tan et al. (2018) measured serum exosomal expression of *HOTAIR* in glioblastoma multiforme cases, in comparison to control subjects. Area under the ROC curve (AUC) analyses for *HOTAIR* expression indicated significant differentiation of the cases from the controls with a high specificity and sensitivity.

### *HOTAIR* as a Therapeutic Target

Several recent in vivo and in vitro studies have proposed *HOTAIR* as a tremendous biotarget for anti-glioma therapies (Angelopoulou et al. 2020). Multiple anti-glioma therapeutic agents/strategies which act by repressing the expression of *HOTAIR* have been proposed. Fang et al. (2016) synthesized magnetic nanoparticles encapsulating siRNAs against *HOTAIR*, and demonstrated them to efficiently repress its expression in glioma cells, resulting in robust retardation of tumorigenicity. The anti-tumorigenic actions of these si-*HOTAIR*-containing magnetic nanoparticles were proposed to be reliant on induction of programmed cell death 4 (PDCD4) in the absence of the *HOTAIR*-EZH2/LSD1 interactions, and the consequent reductions in the expressions of cell cycle regulators, cyclin D1, Ki67, brain-derived neurotrophic factor (BDNF), and cyclin-dependent kinase 4 (CDK4) (Fang et al. 2016). More recently, hyperactivation of *HOTAIR* in glioma pathogeneses has been shown to be associated with histone H3K27-methylation-mediated transcriptional repression of peroxisome proliferator-activated receptor alpha (PPARα). This information was used for design of a combinatorial therapeutic strategy of fenofibrate-mediated PPARα activation and siRNA-mediated HOTAIR repression to robustly retard glioma proliferation and invasion (Zhu et al. 2021). Further, a novel strategy for inhibition of pro-oncological activities of *HOTAIR* has been illustrated by Battistelli et al. (2021). In this study, the authors designed a dominant negative mutant of *HOTAIR*, rendering it incapable of affecting EZH2-PRC2 complex in the normal manner. Mutant *HOTAIR* was found to be effective in abolishment of tumor cell motility, invasiveness, and EMT in a Snail protein-dependent manner (Battistelli et al. 2021). Needless to say, more studies are needed to extend the utility of this method in clinical practice. Perhaps, smart glioma-targeting nanoplatforms (Ahmad et al. 2022) might aid in enhancing the specific targeting of this dominant negative *HOTAIR* mutant to glioma sites in human cases.

*HOTAIR* is also reported to be a key biotarget of oncolytic virus-mediated glioma therapy. Treatment of glioblastoma U251 cells with HSV-G47∆ oncolytic viral particles results in reduced tumorigenicity in complementation with reductions in the levels of prominent cancer-related lncRNAs, including *HOTAIR* (Vazifehmand et al. 2022). Evidence also suggests the utility of clustered regularly interspaced short palindromic repeat-based interference (CRISPRi) strategies in identification and subsequent genetic targeting of tumorigenic lncRNAs such as *HOTAIR* in gliomas (Attenello et al. 2021). Further studies however are warrantied to extend and establish these findings.

Pharmacological studies have also been evaluated/directed to target hyperactivated *HOTAIR* signaling in gliomas. For instance, cisplatin has been illustrated to inhibit EMT of multiple types of glioblastoma and neuroblastoma cells, via its action on *HOTAIR* signaling, among other bio-targets (Gonçalves et al. 2020). Bioactive phytochemicals, such as schisandrin B (Sch B) derived from the medicinal plant *Schisandra chinensis,* have also been proposed to elicit significant anti-glioma repressive actions against *HOTAIR* sponging of miR-125a-mTOR signaling (Jiang et al. 2017). Molecular docking and simulations combined with experimental analyses have identified AC1Q3QWB (or simply AQB) as a potential inhibitor of *HOTAIR*-EZH2-PRC2 signaling. In primary patient-derived glioblastoma cells, AQB was found to robustly repress *HOTAIR* signaling, resulting in beneficial induction of tumor suppressor gene, APC regulator of WNT signaling pathway 2 (APC2), and concomitant suppression of Wnt/β-catenin signaling cascade. In addition, complementation of AQB with 3-deazaneplanocin A (DZNep), an inhibitor of the histone methyltransferase EZH2 resulted in much higher anti-tumorigenic actions, compared to treatment with DZNep alone (Li et al. 2019a). AQB has also been found to induce the expression of tumor suppressor genes, including CWF19-like cell cycle control factor 1 (CWF19L1) which controls cell cycle progression by modulating the stability of cyclin-dependent kinases CDK4/6. Indeed, synergistic actions of AQB and CDK4/6 inhibitor palbociclib were illustrated to induce greater degree of anti-tumorigenic signaling (Shi et al. 2020). In their follow-up study, the research group identified programmed death ligand 1 (PDL-1)-mediated activation of pro-inflammatory nuclear factor kappa B (NF-κB) signaling as a critical *HOTAIR*-based therapeutic target against immune tolerance of gliomas (Wang et al. 2021b). Further, they reported that AQB could significantly attenuate the upregulation of inflammatory mediators in U87 and TBD glioma cells. AQB-mediated inhibition of *HOTAIR* was shown to result in increased immune sensitivity and T-cell toxicity of glioma cells, consequently resulting in tumor load reduction and increased survival of immune-compromised mice challenged with orthotopic glioma transplantation. Mechanistic analyses of these AQB-mediated anti-tumorigenic effects suggested that inhibition of *HOTAIR* signaling may culminate into normalization of the expression levels of ubiquitin regulatory X domain protein 1 (UBXN1) (Wang et al. 2021b). A combinatorial therapeutic regimen of cotreatment with AQB and inhibitor of lysine-specific demethylase 1 or LSD1, GSK-LSD1 has also been propounded as an efficient therapeutic strategy against gliomas (Zhao et al. 2021). Since interaction of *HOTAIR* with EZH2 is reliant on the former’s functional 5′-domain and its binding to LSD1 requires an intact 3′-domain of *HOTAIR*, synergistic application of AQB and GSK-LSD1 is expected to result in robust protection against gliomas. Indeed, this therapeutic strategy has been found to efficiently inhibit the expression of cell cycle progression genes as well as activate pro-apoptotic genes both in vitro and in patient-derived xenograft models (Zhao et al. 2021). Another specific small-molecule inhibitor of HOTAIR-EZH2 interaction has been identified in AC1NOD4Q (*aka.* ADQ) by Ren et al. (2019) using in silico analyses. This inhibitor was found to successfully inhibit H3K27-methylation of NLK, a well-known downstream target of *HOTAIR*-EZH2 signaling, with consequent inhibition of tumor pathogenesis in a Wnt/β-catenin pathway-dependent manner. Further, experimentation based upon RNA immunoprecipitation (RIP) and electrophoretic mobility shift assay (EMSA) identified the binding site of ADQ to 5′-domian of *HOTAIR*, which overlaps with the EZH2 binding site (Ren et al. 2019).

Pharmacological strategies to block the activities of bromodomain and extraterminal (BET) proteins are implicated in the therapy against glioblastoma multiforme (Gargano et al. 2023). Evaluation of the molecular mechanisms underlying the anti-oncological effects of BET bromodomain inhibitor I-BET151 in glioblastoma LN18 cells has revealed the significance of abrogation of hyperactivation of *HOTAIR* lncRNA (Pastori et al. 2015). Indeed, protective effects of I-BET151 were severely compromised by overexpression of *HOTAIR*. Interestingly, bromodomain containing 4 (BRDA4) protein binds directly to *HOTAIR* promoter, further supporting the idea that anti-tumorigenic actions of BET protein inhibitors are mediated, at least in part, via altering *HOTAIR* signaling (Pastori et al. 2015).

## *HOTAIR* in Non-oncological Brain Pathophysiological States

**Fig. 3:**
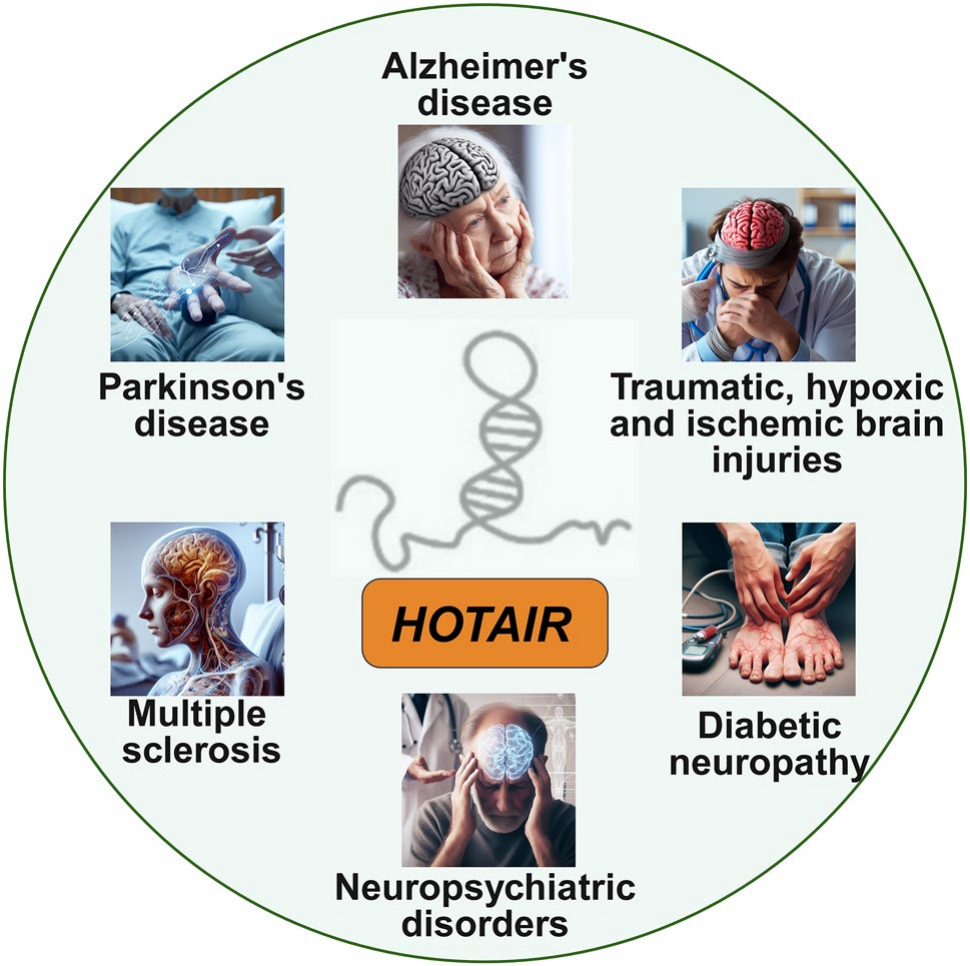
HOTAIR in neuronal pathophysiology. Recent studies implicate deregulated HOTAIR signaling in several non-oncological brain disorders

While multiple studies using cell cultures, animal models and human clinical cases have irrefutably established *HOTAIR* as a prominent pathogenic player in cancers, including gliomas as discussed, a comprehensive understanding of its pathogenic roles in other neuronal disorders has remained largely undiscerned. This is changing as evidenced by the recent surge in studies proposing lncRNAs, including *HOTAIR* as important mediators of different aspects of non-oncological CNS disorders (Fig. 3), such as neurodegeneration, psychiatric illnesses, and developmental conditions (Li et al. 2020; Wu and Kuo 2020; Canseco-Rodriguez et al. 2022). This section first considers the different non-oncological neuronal pathways influenced by *HOTAIR* lncRNA. Next, the evidences for involvement of HOTAIR signaling in the pathophysiology of a plethora of non-cancerous neuronal disorders, including neurodegenerative diseases (e.g., Alzheimer’s disease, Parkinson’s disease and multiple sclerosis), traumatic, ischemic and hypoxic brain injuries, and neuropsychiatric conditions, are discussed (Table 2).

**Table 2:** Summarization of studies implicating *HOTAIR* in the pathophysiology of non-oncological neuropathologies

Pathological state	Disease model	Intervention/study	Pathophysiological effect	References
Alzheimer’s disease	Triple transgenic model of AD (3xTg-AD) mice	Microarray analyses for expressional profiling of lncRNAs	Increased expression of *HOTAIR*	Lee et al. (2015)
Alzheimer’s disease	Human clinical cases	Investigation of *HOTAIR*’s involvement in the regulation of exercise in AD patients	Elevated expression of serum *HOTAIR* and its negative association with cognitive scores; physical activity attenuated these deficits	Lu et al. (2022a)
Alzheimer’s disease	Brain tissue samples of AD patients	Involvement of lncRNAs in multiple layers of CDK5R1 regulation in AD	*HOTAIR*, through its modulation of the miR-15/107 pathway, negatively affected the expression of Cdk5, causing region-specific changes in the brain tissues of Alzheimer’s patients	Spreafico et al. (2018)
Parkinson’s disease	MPTP-mediated mouse model and an in vitro PD model composed of MPP^+^-challenged SH-SY5Y cells	*HOTAIR*’s role in PD-induced MPTP and the possible link between *HOTAIR* and LRRK2 in human neuroblastoma cell lines	Elevated expression of *HOTAIR*, in conjugation with elevated levels of LRRK2 protein	Liu et al. (2016)
Parkinson’s disease	MPTP induced mouse model of PD and an in vitro PD model composed of (MPP^+^)-challenged SH-SY5Y cells	Detection of *HOTAIR*’s expression, miR-221-3p, α-synuclein, and genes associated to apoptosis	Sponging of miR-221-3p mediated by *HOTAIR* directly resulted in overexpression of α-synuclein and its consequent aberrant aggregation, culminating into activation of cellular death pathways	Sun et al. (2022)
Multiple sclerosis	Peripheral blood mononuclear cells (PBMCs) from human clinical cases and mouse model	Evaluation of vitamin D’s role in combination with *HOTAIR* and *ANRIL* lncRNAs using RT-PCR analyses	Significant upregulation of *HOTAIR*’s expression, and confirmation of *HOTAIR* being one of the targets of vitamin D therapy	Pahlevan Kakhki et al. (2018)
Multiple sclerosis	PBMCs from 60 relapse-remitting MS (RRMS) patients	Evaluation of the levels of NF-κB-associated lncRNAs, such as *HOTAIR*, *THRIL*, *H19*, *NKILA*, and *ANRIL*, as well as IL-6, TNF, and MMP9 expression	Increased levels of *HOTAIR* in PBMCs of RRMS patients in the relapse phase	Soltanmoradi et al. (2021)
Multiple sclerosis	Human clinical studies and animal models	Analyses of *HOTAIR* SNPs	HOTAIR SNP rs4759314 correlated with MS etiology in Iranian subjects	Taheri et al. (2020)
Traumatic brain injury (TBI)	rodent model of TBI based on the Feeney’s free-fall impact method, and LPS-challenged microglial BV2 cells	Elucidation of the neuroinflammation-stimulating roles of *HOTAIR* in TBI	Drastic increases in the expression of *HOTAIR* resulting in elevated release of microglial inflammatory mediators, TNF-a, IL-1b, and IL-6	Cheng et al. (2021)
Hypoxia and ischemia	MCAO-mediated hypoxia-induced rodent model and HT22 cell lines	*HOTAIR*’s role in ischemic infarct and its relationship to NOX2 levels during hypoxia-induced ischemic infarct	Hypoxia induction via MCAO culminated into increased *HOTAIR* levels in ischemic infarction lesion sites, and was linked to significant elevations in NOX2	Yang and Lu (2016)
Ischemic stroke	In vivo pMCAO mouse model and an vitro OGD cellular model	Aggravation of stroke pathology in a *HOTAIR*/miR-148p/Kruppel-like factor 6 (KLF6) axis-dependent manner	Involvement of *HOTAIR* in inducing pro-inflammatory and apoptotic signaling in a STAT3-reliant pathway; *HOTAIR* knockdown reduced neurological deficits and cognitive decline	Huang et al. (2021)
Hypoxic-ischemic encephalopathy (HIE)	Neonatal patients with HIE and human brain microvascular endothelial cells (hBMVECs) subjected to oxygen‐glucose deprivation/reperfusion (OGD/R) injury in vitro model	Role of *HOTAIR* in OGD/R‐induced damage in hBMVECs and assessment of serum *HOTAIR* levels in neonatal HIE patients, as well as evaluation of *HOTAIR* effects using in vitro assays	Increased *HOTAIR* expression in models of OGD/R damage and neonatal HIE patients, and reliance of its interaction with EZH2 for aggravation of the dysregulations in cellular migration, membrane disintegration, and pro-apoptotic pathways	Wang et al. (2021a)
Cerebrovascular stroke (CVS)	Human clinical cases with and without chronic hypertension	Comparison of the expression of *NEAT1*, *GAS5*, and *HOTAIR* lncRNAs in CVS patients with and without hypertension, as well as control groups	Reduced plasma level of *HOTAIR* transcripts in CVS cases compared to controls, particularly in hypertensive cases, and a positive correlation between *HOTAIR* and the scores on the Severity scores include The National Institutes of Health Stroke Scale (NIHSS)	Ali et al. (2023)
Neuropsychiatric disorders	Human subjects with various neuropsychiatric disorders	Evaluation of the link between an intronic SNP of *HOTAIR*, rs1899663, and various neuropsychiatric disorders, such as methamphetamine addiction, bipolar disorder types I and II, major depressive disorder, schizophrenia, and ADHD in the Iranian population	The rs1899663 SNP was strongly associated with ADHD risk in allelic, co-dominant, and dominant models	Sayad et al. (2020)
Autism spectrum disorders (ASDs)	Human clinical cases	Genotypic analysis of rs12826786, rs1899663, and rs4759314 SNPs within *HOTAIR* in 427 ASD cases and 430 normally developed children	The rs12826786 SNP in *HOTAIR* was found to be significantly linked with ASD phenotype in allelic and recessive models	Safari et al. (2020)

### *HOTAIR* as a Modulator of Neuronal Pathways

It is interesting to note that even though *HOTAIR* is widely expressed in all tissues, brain has one the highest expression levels. Further, *HOTAIR* expression elicits high brain region specificity and temporal dynamicity (Policarpo et al. 2021). This suggests that *HOTAIR* presumably has important functions in the regulation of multiple aspects of nervous system development and pathophysiology, not just limited to tumorigenicity. Indeed, recent researches have increasingly uncovered multifaceted regulatory roles of *HOTAIR* in neurodevelopment, neuronal signaling and plasticity, and neuroinflammation, as delineated below.

Protein phosphorylation as a posttranslational mediator of spatio-temporal regulation of protein presence, stability, interactions, and function has widespread implications for cellular mechanisms. With regard to *HOTAIR* functions, its interaction with EZH2 has been elicited to be under regulation of the latter’s site-specific phosphorylation status. Thus, binding of *HOTAIR* to EZH2 is enhanced upon its phosphorylation at threonine-345 (T345) (Kaneko et al. 2010). Since EZH2 is critically implicated as a regulator of neuronal differentiation and regeneration pathways (Yu et al. 2013), it is interesting to hypothesize a prominent posttranslational modification-dependent role of *HOTAIR* in neuronal maturation, as well as in neuronal regeneration after insults/injuries. This is supported by evidences that *HOTAIR*-EZH2-miRNA-141 signaling drives expressional changes in BDNF, and regulates the differentiation of dopaminergic neuron-like cells from human amniotic epithelial stem cells (HuAESCs) (Liu et al. 2018b). Along similar lines, Khani‑Habibabadi et al. have recently implicated *HOTAIR* in the regulation of oligodendrocyte precursor cell differentiation (Khani-Habibabadi et al. 2022).

Evidences suggest that *HOTAIR* may also influence protein stability, interactions, and functions by targeting their ubiquitin-mediated proteolysis. For instance, *HOTAIR* has been reported to interact with RNA-binding domains of E3 ubiquitin ligases, DAZ-interacting zinc finger 3 (Dzip3), and Mex-3 RNA-binding family member B (Mex3b), and facilitate ubiquitination and degradation of cellular senescence proteins, ataxin-1 and snurportin-1 (Yoon et al. 2013). Noteworthy, ataxin-1 is a critical modulator of neurodevelopmental cascades and is implicated in various neurological disorders, including spinocerebellar ataxia type 1 (Ju et al. 2014). Also, *HOTAIR*-Mex3b interactions are thought to alter the function of suppressor of mothers against decapentaplegic family member 4 (SMAD-4) and nucleoside diphosphate linked moiety X-type motif 3 (NUDT-3) (Barrios-Rodiles et al. 2005), further supporting the involvement of *HOTAIR* in neuronal physiology.

Mediators of inflammatory signaling, cytokines, and chemokines are generated by microglia, astrocytes, and endothelial cells in the CNS and drive neuroinflammatory responses (DiSabato et al. 2016). Association of hyperactivated inflammatory responses with cellular oxidative stress and damage is well established, and pro-inflammatory and prooxidant signaling have been shown to drive pathogenic mechanisms in many diseases (Chatterjee 2016), including CNS disorders. Contributions of lncRNAs, such as *HOTAIR* to inflammatory and oxidative pathways in CNS pathophysiology are supported by several research studies (Moreno-García et al. 2020; Luo et al. 2021). Hence, *HOTAIR* may drive microglia activation and regulate the release of inflammatory factors (Cheng et al. 2021). Similarly, pro-inflammatory actions of hyperactivated *HOTAIR* have been reported to underlie inflammatory injury, oxidative damage, and cellular apoptosis in spinal cord ischemia–reperfusion injury, possibly in a high mobility group box 1 (HMGB-1)- and NF-κB-dependent manner (Wang et al. 2022c).

### *HOTAIR* as a Pathogenic Player in Non-oncological Neuronal Pathologies

#### Neuropsychiatric Disorders

While the molecular mechanisms have remained undiscerned, clinical studies have indicated probable genetic associations between *HOTAIR* and pathogeneses of psychiatric disorders. Thus, evaluation of rs1899663, an intronic SNP of *HOTAIR* revealed its robust association with bipolar disorder type I in the allelic, co-dominant, and dominant models, bipolar disorder type II in allelic and dominant models, and major depressive disorder (MDD) in allelic and dominant genetic models. The SNP was also found to be strongly associated with the risk of developing attention deficit hyperactivity disorder (ADHD) in allelic, co-dominant, and dominant models of inheritance. However, no links between rs1899663 SNP and the development of methamphetamine addiction or schizophrenia were observed in any of the inheritance models assessed (Sayad et al. 2020). Another clinical study in subjects diagnosed with bipolar disorder (and controls) implicated *HOTAIR* SNPs rs1899663 G/T, rs12826786 C/T, rs4759314 A/G, and rs920778 C/T as risk factors for the development of bipolar disorder under allelic, recessive, dominant, and co-dominant contrasted genetic models (Sargazi et al. 2022). Interestingly, while CT genotype of rs920778 C/T, GT genotype of rs1899663 G/T, and CT genotype of rs12826786 C/T SNPs were proposed to increase the chances of bipolar disorder development, GG genotype of rs4759314 A/G SNP was proposed to attenuate the risk (Sargazi et al. 2022). Autism spectrum disorders (ASDs) are a group of heterogeneous neurodevelopmental behavioral conditions with complex genetic etiologies. In a large-scale genetic study involving 427 ASD clinical cases and 430 normal children, rs12826786 SNP of *HOTAIR* was reported to be significantly associated with ASD phenotype in allelic (T vs. C; odds ratio, OR 1.29) and recessive (TT vs. TC + CC; OR 1.60) models (Safari et al. 2020). Although these studies provide some evidence for genetic association of *HOTAIR* lncRNA in the pathogeneses of psychosocial disorders across multiple age groups, future studies must be directed at understanding the underlying mechanism and players linking the two.

#### Traumatic Brain Injury (TBI)

Comprehensive understanding of lncRNAs as mediators of the pathophysiology of brain injuries, such as stroke and TBI, has been initiated only recently (Zhang et al. 2023). One of the first studies to propose neuroinflammation-stimulating roles of *HOTAIR* in TBI came from Cheng et al. (2021). They employed Feeney’s free-fall impact method for induction of TBI and elucidated drastic increases in *HOTAIR* expression levels, concomitantly with elevated microglia-mediated secretion of pro-inflammatory cytokines, IL-1β, IL-6, and TNF-α. Further, these pro-inflammatory effects of *HOTAIR* were attributed to repressed Nrdp1 (E3 ubiquitin-protein ligase)-mediated ubiquitination and heightened stability of pro-inflammatory protein, myeloid differentiation primary response protein 88 (MyD88). Conversely, silencing of *HOTAIR* was found to normalize Nrdp1 and MyD88 levels and prevent aberrant microglial activation (Cheng et al. 2021).

#### Ischemic and Hypoxic Brain Injuries

In rodent hypoxia models of middle cerebral artery occlusion (MCAO), levels of *HOTAIR* have been shown to be significantly elevated in the ischemic infarction lesion sites. Such increases have been found to be associated with hyperactivation of NADPH oxidase 2 (NOX2), a protein which is thought to drive pathogenic pathways in stroke and ischemic injury to the brain. Further, RNA interference confirmed the roles of *HOTAIR*–NOX2 interactions in induction of apoptotic pathway in hypoxic cells (Yang and Lu 2016). Molecular sponging of specific miRNAs [e.g., miR-211; (Ma et al. 2020)] mediated by *HOTAIR* has also been proposed as a key event affecting the pathology of cerebral ischemia–reperfusion injury. For instance, *HOTAIR*-miR-148p-Kruppel-like factor 6 (KLF6) signaling cascade has been implicated induction of neuronal death following ischemic stroke (Huang et al. 2021). Indeed, hyperactivation of *HOTAIR* and the consequent sponging of miR-148p/KLF6 is observed in both oxygen-glucose deprived (OGD) cells and MCAO mice, resulting in aberrant activation of inflammatory and apoptotic pathways in a STAT3-dependent manner. Further, knockdown of *HOTAIR* in MACO mice was linked not only with increased neuronal survival, but also with significant attenuation in the severity of behavioral, sensorimotor, and proprioceptive deficits (Huang et al. 2021). In accordance with these results, human brain microvascular endothelial cells (hBMVECs) when experimentally challenged with OGD/reperfusion (OGD/R) injury showed significant elevations in the abundances of *HOTAIR* transcripts (Wang et al. 2021a). The consequent hyperactivation of *HOTAIR*-EZH2/PRC2 signaling induced membrane disintegrity and stimulated pro-apoptotic pathways in the OGD/R endothelial cells. Interestingly, these results of involvement of *HOTAIR*-EZH2 axis in propagating blood brain barrier (BBB) deficits are supported by clinical data which shows that there are significant increments in *HOTAIR* levels of plasma samples obtained from neonatal human subjects of hypoxic‑ischemic encephalopathy (HIE) (Wang et al. 2021a). Along similar lines, Ali et al. (2023) have recently evaluated the expressional changes in serum *HOTAIR* among cerebrovascular stroke (CVS) subjects, with and without chronic hypertension, and controls. Interestingly, they reported diminished plasma levels of *HOTAIR* transcripts in CVS subjects, compared to the controls. Moreover, stroke subjects with chronic hypertension were found to have reduced *HOTAIR* levels with respect to those without hypertension (Ali et al. 2023).

#### Parkinson’s Disease (PD)

PD is the most widely studied neurodegenerative condition when it comes to the evaluation of pathophysiological functions *HOTAIR* (Zhang et al. 2022), and a plethora of miRNA species have been implicated as targets in *HOTAIR*-mediated loops of ceRNA networks in PD (Asadi et al. 2023). The pioneering studies linking *HOTAIR* to PD pathogenesis employed chemical N-methyl-4-phenyl-1,2,3,6- tetrahydropyridine (MPTP)-induced in vivo, and *N*-methyl-4-phenylpyridinium (MPP^+^)-induced in vitro SH-SY5Y cellular models. Experimental data from both indicated robust upregulation of *HOTAIR*, in complementation with hyperactivation of leucine-rich repeat kinase 2 (LRRK2), a well-established pathogenic mediator of PD development. Further, siRNA-mediated repression of *HOTAIR* elicited significant neuroprotective effects and resulted in the rescue of LRRK2 dysfunction (Liu et al. 2016; Wang et al. 2017). Aberrant *HOTAIR* signaling in PD has also been linked to altered sponging of miR-126-5p, with the consequent deficits in the activities of RAB3A interacting protein (RAB3IP), a proteinaceous player implicated in synapse development, signaling, and maintenance (Lin et al. 2019). Correspondingly, repression of *HOTAIR* expression and the beneficial changes in miR-126-5p/RAB3IP signaling have been reported to inhibit the loss of tyrosine hydroxylase (TH)-positive cells and reduce the abundances of α-synuclein-positive cells, in conjugation with significant attenuation of PD-associated decline in motor and behavioral functions (Lin et al. 2019).

Other studies have also reported involvement of additional miRNAs as sponging targets of *HOTAIR* in the pathophysiology of PD. For example, Lang et al. (2020) showed that elevated *HOTAIR* expression induces autophagy-related genes (lysosomal-associated membrane protein types I and II; LAMP1 and 2, and microtubule associated protein 1 light chain 3 beta LC3B-I/LC3B-II ratio) in dopaminergic neurons by altering miR-221-3p/neuronal pentraxin II (NPTX2) axis. *HOTAIR* may also promote NLR family pyrin domain containing 3 (NLRP3)-mediated pyroptosis in neurons as a consequence of its sponging actions targeting miR-326 (Zhang et al. 2021). Further, alterations in the *HOTAIR*-miR-874-5p-autophagy-related protein 10 (ATG10) signaling axis and aberrant activation of pro-inflammatory, pro-apoptotic, and pro-oxidative signaling have also been observed in MPP^+^-induced cellular model of PD (Zhao et al. 2020a). More recently, miR-221-3p has been reported as another sponging target of *HOTAIR* linked to overexpression of α-synuclein and its aberrant aggregation in PD (Sun et al. 2022).

#### Alzheimer’s Disease (AD)

The first study to link *HOTAIR* with AD was performed by Lee et al. who evaluated the expressional changes in multiple lncRNA species, and observed significantly dysregulated expression of *HOTAIR* in 3xTg-AD mice (Lee et al. 2015), a genetic model of familial AD which incorporates both amyloid and tau pathologies. Similar results of elevated *HOTAIR* levels were obtained in APP/PS1 mice, another genetic model of AD (Lu et al. 2022a). Interestingly, voluntary exercise in these mice was observed to robustly attenuate pro-inflammatory responses and rescue spatial memory deficits, possibly via normalization of hyperactivated *HOTAIR* and rectification of its sponging actions on the target miR-130a-3p (Lu et al. 2022a). miR-15/107 has also been proposed as a mediator of pathogenic actions of *HOTAIR* in AD (Spreafico et al. 2018). In this study, cyclin-dependent kinase 5 (Cdk5) was identified as a target of lncRNAs; nuclear-enriched abundant transcript 1 (*NEAT1*) and *HOTAIR*. Notably, Cdk5 is a primary focal mediator of tau and amyloid pathologies and is aberrantly hyperactivated in AD. Hence, region-specific alterations in *HOTAIR* expression in brain tissue samples obtained from clinical subjects of AD may contribute to disease pathology via altering Cdk5 signaling (Spreafico et al. 2018). Further, elevated serum expression of *HOTAIR* in AD patients has been reported to elicit close associations with the severity of cognitive and memory impairments (Lu et al. 2022b), encouraging the proposition of serum *HOTAIR* levels as a biomarker for cognitive decline in AD. Interestingly, a 3-month bicycle training paradigm resulted in significant reductions in the serum *HOTAIR* levels in these clinical subjects, concomitantly with robust attenuation of AD-linked cognitive impairment (Lu et al. 2022b).

It should be noted here that while cognitive decline is a chief phenotypic hallmark of AD, it is not a specific outcome of the pathology. Nevertheless, cognitive enhancement is one of the targets of therapeutic strategies against AD. Although not a model of AD, extended exposure to sevoflurane, which is a volatile anesthetic, is known to induce cognitive dysfunction. Wang et al. (2018) showed that exposure of sevoflurane resulted in diminishment of learning and memory functions and BDNF expression in rats. Further, they illustrated that these sevoflurane-mediated detrimental effects were reliant on *HOTAIR*-induced dysfunction of REST. In concurrence, siRNA-induced repression of *HOTAIR* prevented the molecular and behavioral effects of sevoflurane (Wang et al. 2018). Similar results have been observed in isoflurane (another volatile anesthetic)-induced cognitive impairment in rodents. In this study, the authors proposed *HOTAIR*-mediated sponging of miRNA-129-5p as the major pathogenic event contributing to oxidative dyshomeostasis, hyperactivated neuroinflammatory responses, neuronal death, and behavioral deficits (Wang et al. 2022b).

#### Multiple Sclerosis (MS)

*HOTAIR* has also been proposed as a pathogenic player in immune dysfunction and axonal demyelination in MS (Li et al. 2020). In fact, effectiveness of vitamin D3-based therapies in MS patients has been proposed at least in part, to be due to their abilities to normalize the elevated expression of *HOTAIR* lncRNA (Pahlevan Kakhki et al. 2018). Hence, significant rescue of the upregulated levels of *HOTAIR* in peripheral blood mononuclear cells (PBMCs) was observed following vitamin D3 supplementation in MS clinical cases. Vitamin D3-mediated modulation of *HOTAIR* levels was further confirmed in a mouse model of experimental autoimmune encephalomyelitis (Pahlevan Kakhki et al. 2018). Similarly, sulfasalazine, an immunosuppressive pharmacological agent prescribed for MS treatment, was found to significantly decrease *HOTAIR* expression (Duan et al. 2018). Sulfasalazine treatment in mice subjected to chemically (cuprizone)-induced demyelination resulted in inhibition of pro-inflammatory switch of microglial, and induction of oligodendrocyte differentiation and remyelination pathways. Mechanistically, sulfasalazine was shown to repress *HOTAIR* expression, thereby affecting its sponging of the miR-136-5p-Akt2-NF-κB cascade (Duan et al. 2018). Clinical data also support the hypothesis of prominent interference of *HOTAIR* in MS pathology. Thus, it has been reported that rs4759314 SNP of *HOTAIR* is probably associated with increased risk of developing MS (Taheri et al. 2020). Further, increased expression of *HOTAIR* has been observed in PBMCs of relapse-remitting MS subjects in the relapse phase, indicating that it could serve as a potential biomarker to differentiate the remit and relapse phases of these subjects (Soltanmoradi et al. 2021).

#### Diabetic Retinopathy/Neuropathy

Diabetic retinopathy is a serious pathological outcome of hyperglycemia and the consequent damage to retinal cells. Recent studies have provided some evidence for the involvement of *HOTAIR* in diabetic retinopathy. For instance, appreciable enhancement of *HOTAIR* expression has been observed in human retinal endothelial cells challenged with high concentrations of extracellular glucose, and in retinal tissues of streptozotocin-induced animal model of diabetic neuropathy. Hyperactivation of *HOTAIR* was found to be associated with detrimental changes in mitochondrial bioenergetics, redox signaling, and angiogenesis. Correspondingly, RNA interference-mediated repression of *HOTAIR* prevented these hyperglycemia-associated alterations. Further, samples from serum and vitreous humor derived from clinical subjects of diabetic neuropathy elicited aberrantly enhanced expressions of *HOTAIR* lncRNA (Biswas et al. 2021). Clinical data have also illustrated the probable linkages of *HOTAIR* SNPs in diabetic retinopathy. In a case–control study by Chuang et al. (2022) involving subjects with diabetic retinopathy and controls, SNPs rs12427129-CT, rs12427129-CT + TT, and rs1899663-TT were reported to have aberrantly upregulated expression in diabetic retinopathy patients. Moreover, significantly increased presence of SNPs rs12427129-CT + TT and rs1899663-TT were confirmed in clinical subjects of proliferative diabetic retinopathy (Chuang et al. 2022).

## Conclusions

Recent studies clarify the multifaceted roles of *HOTAIR* in neuropathologies. Particularly, its implications as a pro-oncogenic lncRNA are increasingly becoming evident. A plethora of downstream molecular targets and pathways detrimentally affected by *HOTAIR* during the different stages of glioma have been identified. Nevertheless and in spite of surges in research studies, *HOTAIR*’s role in other neuronal pathologies has largely remained obscure. This review is a timely and comprehensive attempt to summarize the available research data implicating *HOTAIR* as an impactful player in the development of multiple neuronal disorders. In doing so, the authors hope that it will serve as a platform for more research studies directed at thorough understandings of the molecular mechanisms underlying *HOTAIR*-mediated induction of pathological events in the brain. Lastly, we anticipate that further studies are directed to evaluate and establish the utilities of *HOTAIR* as a potential biotarget for the design of effective multimodal neuroprotective strategies.

## Data Availability

Not applicable.
